# Molecular mechanisms regulating T helper 2 cell differentiation and function

**DOI:** 10.1016/j.coi.2024.102483

**Published:** 2024-10-01

**Authors:** Aydan CH Szeto, Ana CF Ferreira, Andrew NJ McKenzie

**Affiliations:** MRC Laboratory of Molecular Biology, Cambridge CB2 0QH, United Kingdom

## Abstract

T helper 2 (T_H_2) cells orchestrate type 2 immunity during protective antihelminth immunity and help restore tissue homoeostasis. Their misdirected activities against innocuous substances also underlie atopic diseases, such as asthma and allergy. Recent technological advances are uncovering novel insights into the molecular mechanisms governing T_H_2 cell differentiation and function.

## Introduction

Type 2 immunity helps maintain and restore tissue homoeostasis and protects from parasitic helminth infection. It is principally orchestrated by adaptive T helper 2 (T_H_2) cells and group 2 innate lymphoid cells (ILC2s) and can lead to asthma and allergies when misdirected. T_H_2 cells belong to a subfamily of CD4^+^ T helper lymphocytes that display polarised cytokine production (including interleukin [IL]-4, IL-5, and IL-13) under the control of the master transcription factor (TF) GATA3 via distinct extracellular signals and intracellular pathways (reviewed in Refs. [1,2]). These may vary with the tissue-specific microenvironmental cues encountered, including the cytokine milieu and dendritic cell (DC) interactions, leading to complex intracellular signals triggering dynamic association of TFs, DNA reorganisation and chromatin remodelling (extensively reviewed [2,3]). This review focuses on how new technological advances, including high-throughput CRISPR-Cas9 screens and single-cell gene expression profiling, are increasing our depth of understanding of these processes within the heterogeneity of T_H_2 cell states.

### How and why are type 2 dendritic cells preferentially priming T helper 2 differentiation?

**Figure 1:**
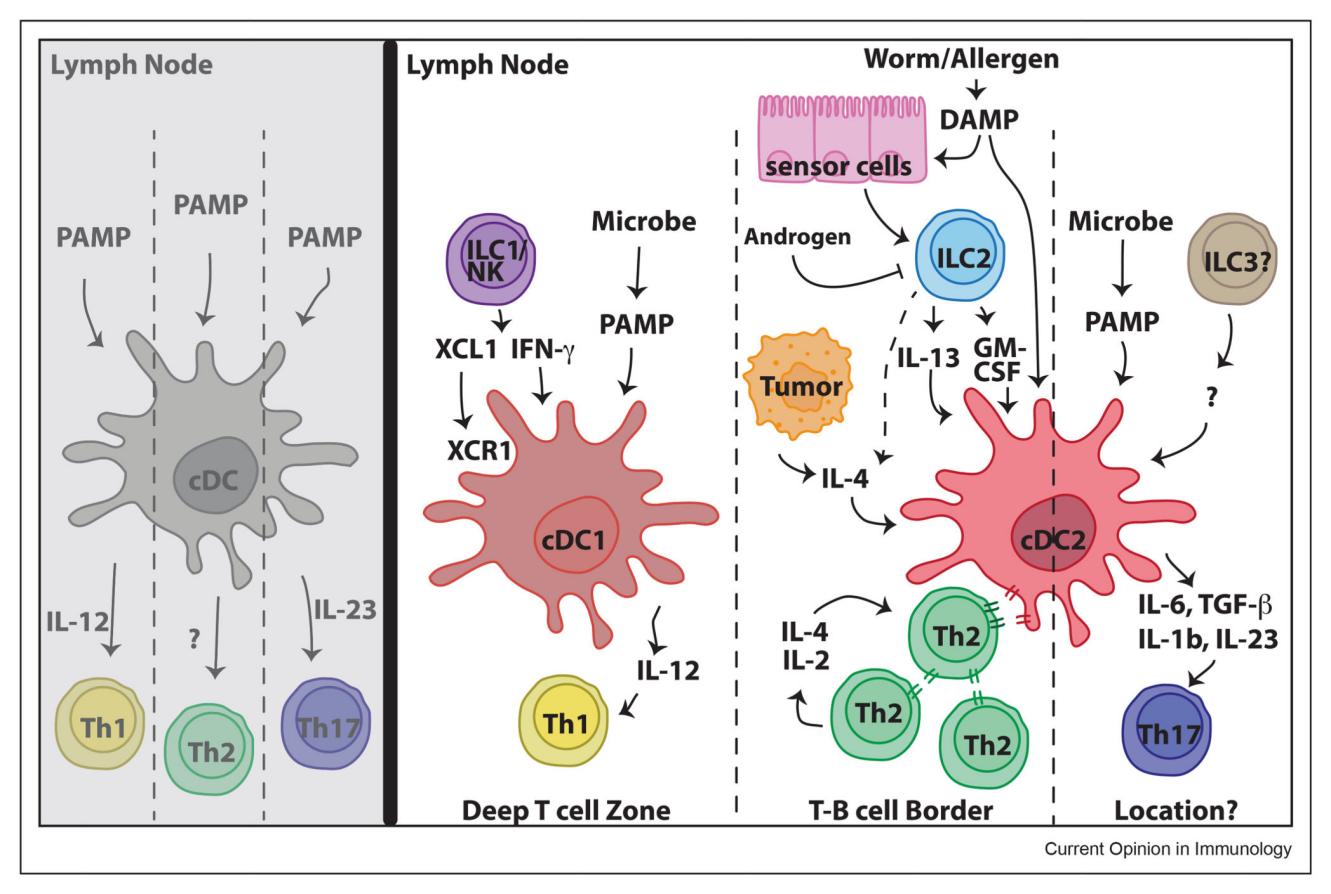
Revised model for the initiation of T_H_2 cell response. Recent discoveries have revealed several properties characteristic of DC polarisation of T_H_2 cell fate, including (1) a distinct DC2 population with reduced capacity to produce the pro-T_H_1 cytokine IL-12; (2) the spatial segregation of DC2s and early T_H_2 cells at the T-B cell border away from the deep T cell zone where DC1-T_H_1 cells reside; (3) the heterotypic and homotypic clustering mechanism among DC2 and T_H_2 cells that reinforce IL-2 and IL-4 signalling within macroclusters; and (4) the modulation of DCs by tissue-resident cells, including ILC2s and other responsive cells and their secreted products. After T_H_2 cell commitment and dissemination to the periphery, T_H_2 cells are subjected to regulation by local tissue checkpoints, including IL-33, IL-25, and thymic stromal lymphopoietin (TSLP), signalling to promote full cytokine production capability (not depicted).

DCs, comprising type 1 (DC1) and type 2 (DC2), are essential players in bridging innate and adaptive immunity by presenting antigens and providing costimulation to naïve T cells. Recent reports have argued for a prominent role for DC2s in specifically regulating T_H_2 cell differentiation [4–7] (Figure 1). This is supported by evidence from mice in which cDC2s are missing [8] due to the mutation of nuclear factor, interleukin 3 regulated (NFIL3)-/CCAAT-enhancer-binding proteins (C/EBP) enhancer binding sites within an enhancer element 165 kb-upstream of the Zeb2 gene, which abolishes Zeb2 expression in the myeloid lineage and prevents DC2 development, leading to impaired T_H_2 responses to *Heligmosomoides polygyrus* infection.

DCs are short-lived tissue-resident sentinels that traffic to the draining lymph node (LN) upon immune activation. It has been proposed that the geographical location of DC subsets is critical for their priming of T_H_2 reactions, with segregation of T_H_1-promoting DC1s deep within the T cell zone of the draining LN, away from the T_H_2-promoting DC2s at the T-B border [9]. More recently, extensive macroclustering behaviour between DC2s and differentiating T_H_2 cells has been directly visualised in the draining auricular LN following cutaneous administration of papain into the ear skin [10]. This macroclustering is dependent on intercellular adhesion molecule 1 (ICAM1)/lymphocyte function-associated antigen 1(LFA-1) and associated with enhanced DC-mediated T cell signalling along the IL-2/signal transducer and activation of transcription (STAT) 5 and IL-4/STAT6 axes [10]. Interestingly, although ICAM1/LFA-1 can also mediate T-T interactions, this specific interaction was not important for early T_H_2 responses as shown by a cell transfer model [10]. However, in a genome-wide CRISPR screen for regulators of T_H_2 function, the expression of the αvβ3 integrin complex was identified to promote homotypic T-T cell interactions and their efficient differentiation during type 2 immune challenge [11]. Integrin αvβ3 is directly and differentially upregulated by IL-4-induced GATA3 in T_H_2 cells and can interact with Thy1 on neighbouring T_H_2 cells to drive homotypic aggregation, mTOR signalling, and potentiate a type 2-polarised microenvironment [11]. Taken together, these studies suggest a model in which initial ICAM1/LFA-1-dependent DC2-T cell macroclusters help define T_H_2 cell fate, which is subsequently amplified by αvβ3/Thy1-mediated T_H_2/T_H_2 homotypic interactions. Both clustering behaviours could contribute to a type 2 permissive environment in which T_H_2 cell−derived IL-2 and IL-4 act in a paracrine manner to more efficiently stimulate and polarise neighbouring T_H_2 cells.

Intriguingly, Lyons-Cohen et al. also reported that papain challenge of different dermatomes (regions of skin which are discretely innervated) elicited distinct outcomes, which are dependent on the differential ability of geographically separated skin DC2s to upregulate costimulatory molecules [10]. In contrast to the response induced in the ear pinnae, papain immunisation in the footpad did not elicit efficient T_H_2 cell responses in the draining brachial LN. This difference correlated with a lower expression of CD80 and CD86 by brachial LN migratory DCs compared to those found in the auricular (ear draining) LN. However, immunisation with CpG, which induces a canonical type 1 response, induced equivalent CD80/CD86 expression by migratory DCs in the brachial and auricular LNs, suggesting that the observed location-specific difference in adaptive immunity is restricted to T_H_2 cell responses [10]. Indeed, the tissue imprinting of homoeostatic signals derived from tissue-resident cell types, for example, ILC2s, has been reported to regulate DC function and diversity in the skin [12–14]. The relative contribution of innate immune cell types in promoting adaptive type 2 immunity appears to be highly tissue and context dependent and is likely to involve multiple accessory cell types (including stromal and immune) to provide the appropriate signals.

Another factor contributing to DC2 functional diversity could be their ontogeny. A recent study identified a lymphoid origin for some, but not all, DC2s in the periphery [15]. Interestingly, both lymphoid- and myeloid-derived DC2s converge to make up the pool of mature DC2s, the proportions of which vary across tissues. Further investigation to explicitly compare lymphoid-versus myeloid-derived DC2s is warranted. Does DC ontogeny contribute to divergent DC2 function, for example, in LN localisation, upregulation of costimulatory molecules, or promoting type-2/17 immunity?

### Tissue checkpoints for T helper 2 cell function

Additional polarising signals from accessory cells act in concert with DC-derived signals 1 and 2 to drive T_H_2 cell differentiation. IL-4 both initiates STAT6 signalling and helps suppress DC IL-12 or IL-23 production (which promotes T_H_1 or T_H_17 fates) [16,17]. While IL-4 is sufficient to drive GATA3 upregulation and T_H_2 fate commitment *in vitro*, other costimulatory signals can contribute, including Notch, OX40L, CD40L, and ICOS [18,19]. The *in vivo* cellular sources of IL-4 (including T cells, basophils, ILC2s, mast cells, and natural killer T [NKT] cells) also vary depending on stimulus and location.

After initial DC-mediated antigen priming of naïve CD4 T cells in the LN, the antigen-restricted T_H_2 cells are sub-sequently exposed to tissue licensing factors in the periphery. For example, stromal cell−derived cytokines, including IL-33, IL-25, and thymic stromal lymphopoietin (TSLP), can potently enhance T_H_2 effector cytokine production. In the combined absence of IL-33, IL-25, and TSLP signalling, initial T_H_2 cell priming in the LN as assessed by IL-4 production remains intact; however, 4get^+^ T_H_2 cells in the tissue are defective in IL-5 and IL-13 secretion [20]. Intriguingly, these tissue licensing factors are also major drivers of ILC2 activation [20], suggesting that once naïve T cells are activated to become primary T_H_2 cells, they converge with ILC2s along a shared programme of regulation. Indeed, Kabat et al. showed that T_H_2 cells, elicited by *H. polygyrus* infection, acquire adipose tissue residency and a transcriptional programme reminiscent of adipose tissue ILC2s, including the expression of *Arg1, Calca*, and *Nmur1* [21]. These tissue-resident T_H_2 cells (T_H_2_RM_) are also activated by IL-33 and TSLP to produce effector cytokines in a T cell receptor (TCR)-independent manner. Such an innate-like mode of T_H_2 cell triggering is likely to contribute to cytokine-driven, antigen-independent ‘bystander’ cell activation, which, together with ILC2s, rapidly responds to immune stimulation but may lead to chronic inflammation where antigen-specificity is over-ridden.

## Intracellular events leading to T helper 2 cell differentiation

**Figure 2:**
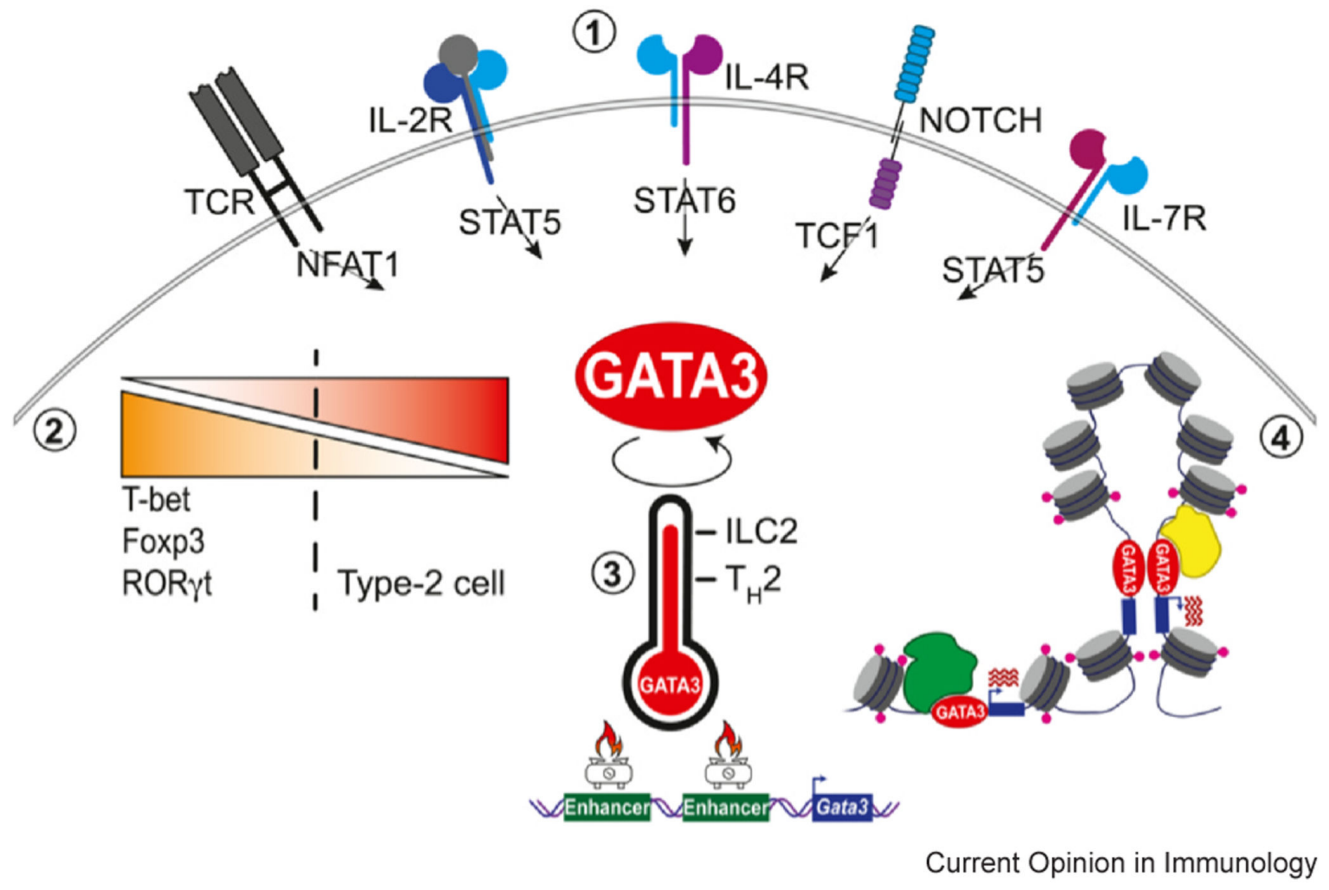
GATA3 as the central regulator of T_H_2 cells. (1) GATA3 transcription is induced by a spectrum of signalling pathways, which can be IL-4 dependent or independent. (2) The equilibrium between master TFs critically determines cell fate. GATA3 expression promotes T_H_2 cell differentiation while repressing the T_H_1 and T_H_17 programme. (3) GATA3 expression is regulated by multiple regulatory regions that have distinct accessibility and impact in ILC2 and T_H_2 cells. Cell type−specific enhancers may ‘fuel’ the differential GATA3 requirements in ILC2s and T_H_2 cells. (4) GATA3 binding to DNA is a key player in the transcription of T_H_2-specific genes while repressing other T_H_ subtypes. GATA3 can associate with distinct protein complexes and promote gene transcription or repression depending on the interaction partners. GATA3 is involved in the formation of chromatin loops and promoting the distinctive chromatin landscape for T_H_2 cytokine expression.

The diversity of extracellular signals is interpreted within the cell via signalling cascades, TF mobilisation, and chromatin reconfiguration, encompassing histone modifications, DNA accessibility, and three-dimensional (3D) organisation (Figure 2).

### Transcription factor balance

GATA3 binding to DNA is a key player in the transcription of T_H_2-specific genes while repressing other T_H_ subtypes [1,2]. The equilibrium between master TFs critically determines cell fate. For instance, ectopic expression of Foxp3 (the master regulator of Tregs) in T_H_2 cells leads to a reduction in GATA3 and type 2 cytokine expression, while Foxp3 knockdown has the opposite effect [22]. Notably, Foxp3 transcription is repressed at an early stage of T_H_2 differentiation preceding the induction of type 2 cytokines [22]. Furthermore, RORγt (the master regulator of T_H_17) can directly inhibit T_H_2 cytokine transcription, with loss of RORγt leading to a decrease in chromatin accessibility at T_H_17-specific gene loci and T_H_17 cell reprogramming towards T_H_2 transcriptional and epigenetic programmes [23].

Hertweck et al. demonstrated that co-expression of T-bet and GATA3 in EL4 cells modifies GATA3 DNA-binding profiles, while T-bet binding remains undisturbed [24]. Interestingly, GATA3 redistribution across the genome is proportional to T-bet levels, with increased T-bet levels leading to the removal of GATA3 from its canonical binding sites, to silence the T_H_2 gene expression programme. Comparison of the orthologous regions of mouse and human genome suggests similar mechanisms may occur in primary human T cells [24].

### Cell type−specific acquisition of type 2−permissive GATA3 expression

GATA3 expression can be induced by diverse signalling pathways. In the context of T_H_2 polarisation, IL-4 is the major driver of GATA3 expression through the activation of STAT6 [2]. Other pathways, including TCR, IL-2, IL-7 [25–27], and NOTCH [28,29], can also promote GATA3 expression.

The requirement for high levels of GATA3 expression is also shared with other type 2 effector cells, including ILC2s. Several recent reports have compared and identified differential chromatin accessibility around the *Gata3* locus between ILC2s and T_H_2 cells [30]. Deletion of ILC2-specific GATA3 enhancers 674 kb downstream of the *Gata3* gene affected ILC2s but not T_H_2 cells [31,32]. The significance of these distinct regulatory regions warrants further investigation but could be linked to the higher GATA3 levels required for ILC2 differentiation. A recent study used GATA3 and IL-13 expression as readouts of ILC2 function in CRISPR screens and identified Mef2d as a TF that potentiates type 2−permissive GATA3 expression in ST2^+^ ILC2s [33]. Mef2d enables optimal GATA3 expression by repressing the *Zc3h12a* locus, which encodes the endonuclease Regnase-1, a known negative regulator of GATA3 and ST2. The Regnase-1/GATA3 regulatory axis appears to be shared between ILC2s and T_H_2 cells, as Regnase-1 mutant mice display hyperactive type 2 inflammation in both cellular compartments [34,35]. T cell−restricted Mef2d-deficient mice also developed lower pulmonary type 2 inflammation, but whether divergent pathways mediate the effects of Mef2d in ILC2s and T_H_2s remains to be addressed [33].

### DNA accessibility and genome architecture

GATA3 is involved in the formation of chromatin loops and reconfiguration of histone H3 lysine 4 methylation (H3K4me1) for T_H_2-specific gene expression [36]. GATA3 deletion reduces the formation of chromatin loops in T_H_2 cells but not naïve T cells [36]. It is known that the spatial interactions of regulatory elements play a critical role in controlling the type 2 cytokine locus, with many regulatory elements described [37,38]. Although techniques allowing single-cell resolution of 3D genome architecture are being developed, current bulk cell analyses make it difficult to accurately appreciate the influence of genome architecture within the heterogeneity of the T_H_2 population. Liu et al. addressed this challenge by separating *in vitro* differentiated T_H_2 cells based on their ability to produce IL-4 [36]. IL-4-positive T_H_2 cells displayed strong interactions between the *Il4* promoter and the type 2 cytokine locus control region (LCR), whereas in IL-4-negative cells, the *Il4* locus was separated from *Il13* and the LCR by a CCCTC-binding factor (CTCF)-mediated loop. This loop forms a physical barrier between the *Il4* gene and the LCR, resulting in the lack of *Il4* expression [36]. In humans, memory T_H_2 cells from peripheral blood also exhibit a spatial genome reorganisation distinct from naïve T cells [39]. Robust and widespread 3D interactions were observed across the type 2 cytokine locus between the LCR and *IL13, IL4*, and *IL5*, which were further augmented upon memory cell activation. Interestingly, naïve T cells have an extra topologically associated domain (TAD) around the *GATA3* locus that isolates it from a downstream regulatory region. This TAD is lost in memory T_H_2 cells, allowing the interaction between *Gata3* and the regulatory element, therefore promoting GATA3 expression [39].

Context-dependent TFs may also play roles in this complex network. For example, deletion of a superenhancer that promotes Ets1 expression allows a T_H_2-like transcriptomic signature to arise in T_H_1 cells [40]. The lower levels of ETS-1 lead to disruption of the T_H_1-specific genome topology through a mechanism involving CTCF recruitment, resulting in enhanced allergic responses [40]. As the type 2 cytokine locus starts to reconfigure for high-level expression of IL-13, chromatin remodelling complexes are recruited for efficient H3K27 acetylation, DNA accessibility, and transcription. One such factor was recently identified in a functional CRISPR screen that revealed a critical role for activity-dependent neuroprotective protein (ADNP) in the recruitment of chromatin remodelling complexes to the type-2 cytokine locus [41]. In the absence of ADNP, T_H_2 and ILC2s fail to orchestrate robust type 2 cytokine responses to lung allergens [41]. This requirement for ADNP extended to other type 2 gene loci, including *cMaf, Il4ra, Itgav*, and *Itgb3*, demonstrating its role in promoting a type 2 phenotype in the lymphocyte compartment [41]. Additional chromatin factors that interact with GATA3 to modify gene accessibility in T_H_2 cells have been recently identified, including Zfp148 and Zfp281 [42].

## Cell-by-cell transcriptomic analysis of T_H_2 diversity in type 2 pathologies

Our understanding of the genes being regulated during the onset of type 2 responses has increased substantially with the development of single-cell transcriptomic analysis [43]. Technological advancements in single-cell RNA sequencing (scRNAseq) enabled the study of patients with various type 2 inflammatory conditions [44] and revealed the heterogeneity and plasticity of human type 2 lymphocytes.

In a comprehensive study [45], a single-cell transcriptomic atlas was curated by combining scRNAseq data in the context of multiple chronic type 2 inflammatory diseases (e.g. asthma; allergic conjunctivitis; chronic rhinosinusitis with nasal polyps [CRSwNP]; ulcerative colitis; atopic dermatitis; bullous pemphigoid; lichen planus; eosinophilic esophagitis; and lymphedema). Thirteen distinct clusters of GATA3-expressing lymphocytes were present across all diseases, including effector cells, T_reg_ cells, and a memory-like subset, revealing a common signature of GATA3-expressing populations across tissues with type 2 inflammation [45]. By combining scRNAseq and TCR sequencing from CRSwNP tissue, the authors assessed the clonal overlap rate between different GATA3^+^ cell clusters and reported a developmental relationship between effector T_H_2 cells, T_FH_, Tregs, and a T_H_2 cell multipotent progenitor (T_H_2-MPP). These observations are consistent with plasticity between some of the GATA3^+^ T_H_ cell subsets, though plasticity between GATA3-expressing and nonexpressing T cells was not addressed. T_H_2-MPP cells were abundant in tissues from chronic type 2 inflammatory diseases and have the potential to sustain type 2 inflammation by self-renewing and differentiating into cytokine-producing effector cells. They were characterised by the lack of CRTH2 expression (CRTH2^−^GATA3^+^ T_H_2) [45]. In human studies, CRTH2 is traditionally used as a marker for T_H_2 cells and ILC2s, especially in circulating cells where the correlation between cytokine production and TF expression is less apparent compared to mice. Thus, the application of unbiased scRNAseq analysis enabled the identification of this previously unappreciated CRTH2^−^ or low T_H_2-MPP population.

Similar observations were reported from additional recent studies on scRNAseq profiling of allergic individuals [46,47]. Dog allergen-specific T cells from allergic donors contained a population that lacked CRTH2 (referred to as T_H_2-like cells) and shared clonal relationships with pathogenic effector T_H_2A cells [46]. In a study involving allergic rhinitis patients undergoing sublingual immunotherapy (SLIT), a differentiation trajectory from CRTH2^−^ T_H_2 cells to Tregs was found to be induced by SLIT and associated with good response to the immunotherapy [47]. This study demonstrates the predictive and potential therapeutic values of CRTH2^−^ T_H_2 cells in human type 2 pathologies revealed by scRNAseq combined with TCR sequencing.

These studies [45–47] support a model in which a stem-like, less activated CRTH2^−^ T_H_2 cell population sustains chronic type 2 inflammation by acting as a source of type 2 effector cells. Interestingly, their expression of TCF7 and LEF1 [45] is reminiscent of stem-like memory CD8^+^ T cells during chronic infections and cancer [48]. CRTH2^−^ ILC2s were also reported in the circulation and lung of asthmatic patients [49,50]. These CRTH2^−^ ILC2s appear to mirror CRTH2^−^ T_H_2 cells, expressing lower levels of type 2 cytokines and, when expanded *in vitro*, showing self-renewal and differentiating into a CRTH2^+^ cytokine-expressing mature form [49,50]. The convergence of shared ILC2/T_H_2 phenotypes and potential regulatory mechanisms in mice (discussed above) and humans may represent novel strategies for therapeutic intervention, for example, to strategically ablate inflammatory progenitors or suppress local inflammation by modulating tissue-derived factors.

Gene expression studies also provided insights on the importance of DCs in modulating T_H_2 cell function in human type 2 pathologies [51,52]. ScRNAseq and mass cytometry analyses of the nasal mucosa from healthy individuals and patients with chronic rhinosinusitis indicated that a DC2−T_H_2 axis, but not a DC1−T_H_2 axis, is characteristic of eosinophilic CRSwNP (eCRSwNP) [51]. More specifically, they identified a LAMP3^+^ DC subset specific to eCRSwNP that expresses genes associated with the migration and recruitment of T_H_2 cells [51]. A study directly comparing allergic asthmatics with allergic nonasthmatic controls showed a cross-talk between IL-9-expressing T_H_2 cells and DC2s in asthmatic airways [52]. These gene expression studies suggest the existence of DC2−T_H_2 interactions within the tissue, which promote DC maturation and T_H_2 costimulation, inducing the pathogenic T_H_2 phenotype in humans [51,52]. Thus, the question remains open: do tissue DC−T_H_2 interactions actively play a causative role in initiating/sustaining type 2 pathologies, or are they merely a downstream signature of already established inflammation? Experimental evidence for the biological importance of this interaction in inflamed tissue is lacking, and future mechanistic studies will be required to clarify whether tissue DC−T_H_2 interaction represents a biologically important therapeutic target.

### How can mouse and human studies inform each other?

As discussed above, recent technological advances have provided valuable insight into the role of type 2 lymphocytes during disease and revealed, perhaps not surprisingly, nuanced differences between mouse and human immune systems. For example, TF co-expression among T cell subsets and T cell lineage plasticity appear to be more prominently observed in humans compared to mice. In humans, but not mice, the presence of type 2 polarised CD8^+^ T cells (Tc2) cells and the putative T_H_2 cell progenitor sustaining type 2 pathologies as discussed above are described. These differences could result from genuine evolutionary divergence or alternatively the current experimental models and/or sterile laboratory environment may not permit the study of analogous cellular processes in the mouse. However, the inclusion of more enriched (humanised) microbiota and the application of more complex/chronic allergen/pathogen challenges should help to better model the human immune system. Nevertheless, the ability to address causal relationships using mouse models will continue to help to provide mechanistic insights where studies in human systems are more difficult to achieve. Recent developments in human immune organoids self-assembled from tonsil and/or spleen cell suspensions may represent an intermediary approach to bridge findings between mouse and human systems [53].

### How can we specifically and functionally investigate the subsets of subsets?

As evidenced above, single-cell technologies have provided remarkable insight into the diversity of cell types, which were traditionally thought to be homogeneous. This diversity may relate to factors including tissue-specific functions or specific pathogen-restricted cell subsets both in the context of antigen-primed T cells (T_H_2), DC subsets (DC2s), and cytokine-secreting helper cells (ILC2s). Whilst illuminating, this information has created new challenges as to how we assign functional importance to these subsets of subsets during complex physiologically relevant immune responses *in vivo*. This problem is especially acute where no subset-specific Cre-driver genes exist to target each individual cell subset without concurrently leading to collateral effects on other related but discrete subsets. The answer may lie in the development of next-generation mouse models, which use intersectional gene expression patterns to drive multiple DNA recombinases or split recombinases [33]. These could be employed to target specific lymphocyte subsets or DC or stromal cell sub-types, to mark or ablate them, or more accurately mediate cell subset gene deletion across tissues and immune challenges.

## Considerations for future studies

As discussed above, the acquisition of full T_H_2 cell effector function is subjected to regulation by cell types and molecules spanning different anatomical locations with inter-related roles. Location-dependent cell labelling technologies such as photoconvertible reporter alleles, and/or tissue-restricted ablation of cell types of interest, may provide greater detail on the spatial aspects of T_H_2 cell differentiation during ageing, obesity, infection, and inflammation. Indeed, an additional layer of cellular complexity may be tissue-specific neuronal subpopulations. Increasing evidence points towards an upstream role for Trpv1^+^ sensory neurons and their release of substance P in initiating dermal type 2 immunity [54,55]. In addition, T_H_2 cells [21] and ILC2s express the receptor for neuromedin U, a neuropeptide produced within the enteric nervous system [56]. The influence of distinct neuronal subsets in different models of type 2 immunity, including protective antihelminth responses, remains to be addressed.

## Summary

The application of single-cell techniques is starting to provide greater detail of the cellular and molecular signals which initiate T_H_2 cell differentiation and their imprinting within the tissues. This now includes new insight into how specific DCs provide the antigen-associated signals to prime the T cells, the stromal factors that additionally guide tissue-restricted patterns of cell specialisation in the context of innate cell help, and how these complex signals are decoded within the nucleus in the context of TF activity, chromatin modifications, and genomic 3D reorganisation. We are now moving from studying average T_H_2 cell responses across heterogeneous cell populations towards being able to discriminate dynamic genome changes, transcriptional profiles and protein production within individual DCs, T_H_2 cells, and costimulatory stromal and immune cell populations. Such data-rich studies encompassing various disease states, over time courses, should provide new opportunities for manipulating the system during discrete disease states to promote and retain advantageous features (e.g. tissue repair) whilst more precisely targeting unfavourable traits at distinct mucosal sites associated with type 2−mediated disease.

## Data Availability

No data were used for the research described in the article.
